# A Monograph on Ovarian Tumors; with an Extended View of Ovariotomy as a Means of Cure

**Published:** 1857-04

**Authors:** T. M. Tweed

**Affiliations:** Eckmansville, Ohio


*A Monograph on Ovarian Tumors; with an extended view of Ovariotomy as a means of cure.* By T. M. Tweed, of Eckmansville, Ohio. (*Continued from page* 419.)

*The Symptoms of Ovarian Dropsy.* We may mention in passing, that the term, Ovarian Dropsy, is applied to almost all cystic tumors, whether they arise in the ovary itself or of its broad ligament, in the omentum, or in the dilatation of the Fallopian tube. They each produce nearly the same symptoms, and, therefore, are only treated separately in the pathological consideration of the subject.

We shall refer the symptoms of this disease to two periods of its progress; the other after it has ascended into the abdominal cavity.

The first symptoms usually brought under the notice of the patient, are a deep seated pain in one or both groins, with great uneasiness; a bearing down and sense of weight in the pelvis; and a peculiar fullness in the lower part of the abdomen. More particular inquiry elicits that there is a throbbing pain almost constantly felt in the fundament; that the pain is produced in passing feces; and that there is also a pain and burning sensation just under the hip of the affected side; most usually the limb on that side becomes numb; a partial loss of motion may occur; pricking and shooting pains are felt, and œdema may take place. The veins of the limb become large in some cases, and piles are often complained of, producing occassional hemorrhage. The menstrual flow is usually regular, but may become profuse, and at this time it often contains white exudations. There is, also, pain in the sexual embrace.

The *touch* at this stage of the disease reveals the uterus healthy and in its natural position; but by placing the finger posteriorly, and at the upper portion of the vagina, the patient will complain of pain, and on further examination, a tumefaction will be found. This is more distinctly felt, and the pain is more acute when the examination is made per rectum.

To the fact of an inflamed ovary being felt through the rectum, and that without difficulty, my own experience abundantly testifies; although this point has been disputed by an eminent Professor of Obstetrics. I have carefully watched the changes that have occurred during the progress and cure of these inflammations, and have distinctly traced the increase and decrease of the diseased organ through the rectum.

Constipation is a most frequent symptom. The pressure of the enlarged ovary on the rectum, and the fear of pain in defecation, soon produce accumulation of feces, which causes distension of the abdomen, not so much, however, from the quantity of the fecal matter retained, as the flatulency produced; so that in the early stages of the disease, a large and flatulent abdomen is usually present, and the patient always complains of distension. The functions of the bladder become interfered with; there is frequent desire to make water, and sometimes inability to pass it.

At the commencement of the disease, constitutional symptoms often arise which simulate those of pregnancy. The stomach frequently becomes affected in the morning; there is occasional syncope; the breast become painful, and it is said that the one on the affected side is more so than the other. These symptoms are all worse at the menstrual period, which in the majority of cases, is but little disturbed, and until the latter stages, is regular, unless the cause of the disease arises from its suppression. At this time a tumor can be felt bulging into the vagina, most usually situated between it and the rectum.

When the tumor rises into the abdomen most of the symptoms we have referred to are alleviated, but others succeed. When the tumor is in the pelvis, it may remain stationary and give little or no trouble to the patient, and is only ascertained when it presents an obstacle to parturition. But, however, it most usually continues, sometimes gradually, at others, very rapidly to increase, and the symptoms at this period are mostly referable to pressure. The bladder now suffers most; its cavity is drawn up, reduced, and unable to distend, so that there is frequent desire to pass water, while small quantities only are ejected. If the tumor occupies the entire abdominal cavity, suppression of urine may result from the pressure of the cyst upon the kidneys. Diuretics fail to increase the quantity of urine, while paracentesis may produce an instantaneous flow, with great relief to the patient. This fact is well illustrated by the case given by Burns of Madame de Rosney, "who in the space of four years, was tapped twenty-eight times; for seven days after each puncture she made water freely, and in sufficient quantity, the appetite was good and all the functions well performed; but in proportion as the tumor increased, the urine in spite of diuretics, diminished, and at last came away only in drops."

When the tumor first occupies the abdominal cavity, there is great tympanitis, so much so as to obscure frequently the disease. We are unable to feel the tumor, and fluctuation can not be perceived; but it gradually increases, displaces the flatulent intestines, and occupies the greater part of the anterior portion of the cavity of the abdomen, causing pressure upon all its organs, and especially upon the stomach, giving rise to frequent sickness after taking food. Edema occurs in the lower extremities, from pressure of the veins of the abdomen; dyspnea comes on from its encroachment on the diaphragm, and the patient gradually sinks from exhaustion.

On examining the abdomen we find a circumscribed tumor in one or both groins, movable under the integuments, not very painful on pressure, and traceable into the pelvis. There may or may not be sensible fluctuation. As the tumor increases it rises above the intestines and stretches the walls of the abdomen, which have a tense and shining appearance, with enlarged veins on the surface. When the patient is lying on her back, the tumor is more apparent on one side than the other. The abdomen, however, retains its prominent shape, is not flattened at its most salient point, and this appearance does not vary, whatever position the patient may assume. The tumor may extend upward to such a degree as to nearly dislocate the ensiform cartilage.

Fluctuation is frequently observed distinctly throughout the whole tumor, which indicates that the fluid is contained in one cavity; while sometimes there are only partial fluctuations, bringing us to the conclusion that it is contained in many. This has been repeatedly observed after death. In a cyst containing two or more cavities, we are unable to recognise fluctuation when the septum intervenes, although it is quite distinct in the individual sacs.

Again, fluctuation may not be perceived so distinctly when any portion of a solid mass intervenes between the fluid and the hand. The mode of testing the presence of solid matter in any portion of the cyst, is to place the hand on one side of the tumor and tap it smartly with the other at a point directly opposite, noting the force of fluctuation; the operation is then to be reversed, and if the fluctuation is equally distinct in both operations we may say there is no solid matter between those points; but if it is less distinct or distant in one case than in the other, the solid matter does exist.

One may be much deceived by the character of the fluctuation, and it becomes a matter of great tact to draw any inference from it. In the first place, a cyst may exist when the fluctuation is very distinct in parts, extending to the circumference of the tumor, and yet it may be composed of numerous small cysts. A case of this kind was seen by many medical men well conversant with the disease, who decided that there was a very large cyst containing solid matter at various points of its inner surface, but the distinct fluctuation was so marked that they supposed the original cyst to be a very large one. This patient submitted to extirpation, and it was found that the whole tumor was composed of an immense number of cysts, and nearly all were filled with a thick fluid. Another case is recorded in which fluctuation was so distinct that all who examined it, decided that it was unilocular in character. She was tapped, but the operator was obliged to desist, for no fluid escaped, though a large trocar was used. She died, and the tumor was found to be composed of a number of cells filled with a thick glairy mucus.

Movements in the abdomen are frequently felt by patients laboring under encysted dropsy. These sensations are variously described, some observe only a beating in the abdomen, others perceive motions like those of a child; but they are all greatly influenced by the mind, and become more frequent the more the attention is directed to them. They usually depend upon the pulsations of the aorta on the distended cyst.

Percussion gives good in ovarian dropsy. There is always dullness from the pubis upward to the circumference of the tumor. This is caused by the mass being placed before the intestines, so that the resonant sound cannot be elicited though it; but if you apply the same means of diagnosis posteriorly in the lumbar regions when the patient is lying on her back, or even around the circumference of the tumor, you obtain a resonant sound; because the intestines occupy those spaces. This is one of the chief distinguishing marks between this disease and ascites; for in the latter when the patient is lying on her back, the fluid gravitates posteriorly, and therefore yields a dull sound in the lumbar regions, and as the intestines are free, they float on the surface of the fluid, and give a resonance anteriorly. On placing the hand gently on the abdomen and carefully moving the parietes on the tumor, we may sometimes feel a crepitus, or a sensation like that produced by the creaking of leather. This is perceived generally where adhesions exist between the tumor and walls of the abdomen, they being long enough to allow of partial motion. But adhesions often exist, where no crepitus can be felt. This physical sign was pointed out by Dr. Bright, who described a case in which it occurred in Guy's Hospital Reports.

The tumor itself is not always found to be smooth in all parts, but frequently portions project and feel hard and solid to the hand, and if firm pressure be used in different parts, hard portions can be ascertained to exist although they do not project. "In this way, if the abdomen be not very tense," says Dr. Bright, "we discover considerable masses of unyielding matter partaking of the general rounded feel of the whole disease, but conveying the impression of more or less flattened spherical bodies attached to the inside of the fluctuating tumor; and these bodies are sometimes so large and so variously placed as to suggest to the unexperienced observer, that the liver, spleen, or the kidneys are enlarged or in some way involved in the disease.

One of the most ludicrous if not serious errors in the diagnosis of this disease which its whole history affords, occurred in the State of Ohio, not many years since. A surgeon of high attainments, extirpated from the side of a woman what he pronounced to be the *Liver.* The Doctor and his colleagues who witnessed the operation, believed it to be so, and published it to the world as one of the most remarkable achievements in surgery! The woman died shortly after, and a *post mortem* examination revealed the fact that there was a healthy and perfect liver still in the abdomen; and the discovery was thus made, that instead of a Liver, they had removed a tumor somewhat resembling that organ; in all probability an ovarian diseased mass.

We frequently find that the vagina is much elongated, giving a funnel-shaped character to the cavity; the os uteri is displaced, usually drawn upward to the side affected, and as it were, twisted upon itself. The uterus is found to be movable and light, and unconnected with the surrounding structures. It can be thrown upon the rectum by the uterine sound; and if the tumor be free, it can be elevated above the pubis. Sometimes, however, the uterus is so pressed between the tumor and the pubis, that it becomes fixed, so that it cannot be moved, and appears connected with the tumor.

The ovarian tumor may project upon the vagina and press together its walls so that it is almost impossible to introduce the finger, or find the os uteri. In one case of this sort which came under our observation, the os uteri was carried quite above the pubis, and with the utmost difficulty could be reached. In some cases the uterus lies across the vagina with its fundus on the rectum and below the sac, so that the finger immediately as it enters the os externum, touches the posterior wall of the uterus. These malpositions can be distinctly ascertained by the uterine sound.

*The Diagnosis of Ovarian Cystic Tumors.*—In no disease is a correct diagnosis of such vital importance as in ovarian dropsy; upon it depends the chance of cure, or the entire abandonment of the patient. Its *difficulty* only equals its *importance;* and to prove this assertion, it is only necessary to refer to the *six* patients who have already submitted to the abdominal section, without saying more of the woman who had her *liver* extirpased—although it proved to be a mistake!

We shall, therefore, so far as our limited opportunities for observation will justify, lay down the diagnostic character of this disease, and point out certain signs whereby it may be distinguished from other abdominal enlargements.

I. When the disease is situated in the pelvis, and before it rises into the cavity of the abdomen, it may be confounded with two malpositions of the uterus, viz: retroversion and retroflection.

1. The ovary is generally felt in the first stage of the disease, between the rectum and the vagina: in some cases fluctuation may be observed, but in others, they are more obscure, and may be mistaken for *retroversion* of the womb. When we examine a patient laboring under the first stages of ovarian dropsy, we find a circumscribed tumor at the posterior portion of the vagina, between that canal and the rectum, painful on pressure, and very much resembling the fundus of the uterus; there may be retention of urine and constipation; the os uteri will be found in its proper position, looking backward, the body of the uterus forward, and movable, as indicated by the uterine sound. The local symptoms are much less severe than if actual retroversion had ocucrred. In retroversion, the os uteri is thrown forcibly forward and upward, and the womb is fixed and very painful.

2. *Retroflexion* of the womb is more likely to be mistaken for cystic dropsy. This is a malposition in which the fundus uteri alone is thrown back upon itself like a common retort, the os being in its natural position, with a tumor directly behind it, between the vagina and rectum. This is the body of the uterus. In such a case, by careful manipulation, the fundus can be restored to its proper position, and the tumor consequently disappears. This will not occur if it be an ovarian tumor.

II. When the tumor occupies the abdomen, it is to be distinguished—

1. *From Ascites.* In ovarian dropsy we find the patient generally in good health, complaining of nothing but the distention of the abdomen, which she states has been gradually increasing, and had its origin in one or both inguinal regions. The abdomen has a tense appearance, and a circumscibed fullness can be perceived. Sometimes, however, the tumor occupies and distends the abdomen to such an extent that this characteristic is not distinguishable. The abdomen is more prominent at one side than the other, and the veins are very much distended. When the stethoscope is applied, no borborigmi are heard, or at least they are heard very indistinctly, while percussion elicits a dull sound anteriorly. But at the upper portion of the tumor, and in the lumbar regions, the clear sound of the intestines is found. Fluctuation is often distinct, but sometimes obscure; more distinct in some parts than others, and is not observed in the lumbar regions.

The dullness on percussion, and fluctuation, are always present *in the same part of the abdomen*, whatever position the patient may assume. They are both found anteriorly. The tumor is usually smooth on its surface, but occasionally irregularities are observed at different points of the abdomen, which appear like projections. These may be so large as to be mistaken for enlargements of the viscera, when in their position; but a careful examination will distinguish these prominences as parts of the cyst. The vagina has a funnel-shaped character, and is usually elongated; the os uteri is turned to the side affected; but sometimes the tumor bulges into this canal, and fluctuation can be felt there.

On the contrary, in *ascites*, the patient has generally a diseased aspect; the abdomen is not tense, and its greatest distention is below, when in the erect posture; on lying down it becomes flattened, change of posture producing change of form. Borborigmi can be distinctly heard with the stethoscope; and when in the supine position, anteriorly percussion elicits a clear resonant sound, which is the result of the intestines floating upon the fluid contained in the cavity. There is dullness in the lumbar regions from the fluid descending to these parts. In the erect posture fluctuation is most distinct anteriorly; in the supine, in the lumbar regions; œdematous effusions are found in other parts of the body, as in the legs; and the veins of the abdomen do not attain so large a size as in ovarian dropsy.

Thus, then,—from the fixed character of an ovarian tumor—from dullness on percussion, anteriorly, not being changed by change of posture—from the resonance in the lumbar regions being constant, and from the tension of the abdominal walls being invariable—we can distinguish cystic dropsy from ascites.

When, however, ascites is present with an ovarian tumor, as frequently happens, the signs of the two diseases become blended. In that case, *slight* percussion produces a superficial fluctuation, seen to the eye, and apparently very near the surface; but if the fingers be suddenly applied to the abdominal walls, they come at once in contact with a hard substance, which evidently gives a fluctuation entirely distinct from the former.

2. *From Pregnancy.* Ovarian dropsy may be accompanied by many of the symptoms of pregnancy. The breasts may become enlarged, and a thin secretion take place; they may become tense and painful, more particularly the one on the affected side. Movements are, also, frequently observed in the abdomen resembling those of a fetus, and the patient may consider herself pregnant. But Ovarian dropsy may be distinguished from this natural state by the disease commencing on one side; by the regularity of the menstrual function; by the absence of the areola and follicles of the nipples; and by the *touch,* which reveals the uterus small, movable, and of its natural form. No ballottement can be perceived, and the os and cervix are of their natural form and consistency. The *bruit* of the enlarged vessels of an ovarian tumor, has been mistaken for the placental murmur, but there is no fetal pulsation. We should recollect, however, that pregnancy may co-exist with ovarian dropsy, and materially obscure the diagnosis. Many women have conceived and borne children during the progress of this disease. The tumid state of the nipple—the areola and follicles—the suppression of the catamenia, the changes which take place in the os and cervix uteri—reveal the presence of pregnancy during the first months; at a later period, the placental murmur and the pulsation of the fetal heart, will dispel all doubts.

3. *From other Cystic Tumors.* It is exceedingly difficult to distinguish these tumors from ovarian dropsy; they assume all the characteristics of the latter disease. We may be assisted when they are small, and if we ascertain their seat of origin; but when large it is impossible to distinguish them by the ordinary means of diagnosis. The uterine sound is the only instrument by which we can distinguish ovarian cysts from other cysts of the abdomen, and this not by positive information, but by negative signs.

"I have found," says Dr. Simpson (London and Edinburg Monthly Journal, for July, 1843,) "advantage from the negative information given by the bougie, even when the tumor was abdominal in its seat. An example will best illustrate my meaning. In a case sent to Edinburg a few months ago, for the purpose of having some opinion given in regard to its nature, an immense abdominal swelling that was present, and which had been supposed by some medical gentlemen who had seen the patient, to be ovarian, was shown not to be so by sufficient evidence of the following nature. The uterus was displaced obliquely backwards, and the fundus of the bladder was displaced to the right illiac region by the abdominal enlargement, circumstances which were easily ascertained by introducing the uterine sound into the cavities of both these organs. Further, the uterus, although displaced, was quite movable, and when its fundus was turned by the bougie toward the site of either ovary, and the abdominal tumor lifted up as high as possible toward the epigastrium, no obstruction was met with: nor was this great change upward in the direction of the tumor found to produce any dragging effects upon the uterus, as held by the bougie or its connections; effects which, unless under the improbable supposition of a pedicle several inches long, would have inevitably occurred if the diseased mass had originated on, or was connected with, the ovaries or uterine appendages. So far the evidence was negative, but so far important. I may add that other characters of a more positive nature—the history, the particular form, consistence of the tumor, its position in point of the substance as ascertained by percussion, etc., seemed to show, seeing that it was not ovarian, it to be in all probability one of those hydatigenous tumors that sometimes form in the tissue of the omentum, and whose physical symptoms during life, in many respects correspond with those of ovarian dropsy.

4. *From Tumors of the Uterus.* These, when they assume the pedunculated form, are very likely to be mistaken for the disease under consideration. Uterine tumors have been removed under the impression that they were ovarian, by Messrs. Heath, Otter, Atlee, and others. A distinguishing sign of these growths is the increased weight of the uterus. If we fi[illegible]d that the uterine sound passes, as it were, into the morbid mass—if the tumor and uterus can not be separated—and if every elevation and depression of the tumor corresponds with the movements of the sound, we may then conclude that the tumor is uterine. But if we find the uterus small and movable, and the sound passes anteriorly to the tumor—if the uterus can be separated from the tumor, and thrown upon the rectum, then we may very confidently state that the growth is not uterine in its character.

5. *From a distended Bladder.* This is not a frequent but an occasional mistake, produced by want of care in the examination. The true state of the case can be readily ascertained by simply introducing a catheter into the bladder—an operation which ought always to be performed before any other is commenced in the organs of the female pelvis. The uterus sometimes, though rarely, is distended with fluid, and may also be mistaken for cystic dropsy. When, however, it is distended to any extent, its tissue is developed as in pregnancy, the cervix disappears, and on percussion of the abdominal parietes, fluctuation can be perceived by the finger in the vagina.

6. *From Carcinomatous and Fibrous Tumors.* In the early stages, ovarian dropsy assumes the hardness of the carcinomatous tumor of this organ; but the latter more rapidly effects the health of the patient. Such tumors are heavier, the surface is much more lobulated, and there is no fluctuation; their progress is very rapid, but they never acquire the size of ovarian cysts.

Again, ovarian dropsy may be mistaken for fibrous tumor. This error of diagnosis may occur in the multilocular variety, complicated with solid matter. A case in point came under the notice of Dr. T. S. Lee. "A patient," says Dr. Lee, "presented herself with an abdominal swelling on the right side, hard and without fluctuation, not at all movable. but it could be traced down into the pelvis; it had been a considerable time in its formation, but it now began to increase. The examination per vaginam, discovered that the brim of the pelvis was occupied by a solid tumor; a small nodule was felt rather in front of the centre of the pelvic cavity, in which was the os uteri. The sound passed upward and forward nearly four inches; it moved with difficulty, as through a cavity, the sides of which were much compressed. This examination was made in December. Here, then, you have every characteristic of a fibrous tumor in the posterior walls of the uterus. The cavity is elongated, the uterus is fixed by the brim of the pelvis, and the tumor in the abdomen is hard and smooth, possessing no fluctuation. The tumor now rapidly increased, and in the January following had occupied the whole cavity of the abdomen. There was then distinct fluctuation in particular parts, and this fact disclosed the real nature of the case, viz: that it was a multilocular cyst, complicated with much solid matter." This is an interesting case, and deserves attentive study.

7. *From accumulation of air in the intestines.* Many instances are on record in which flatulency of the intestines has been mistaken for ovarian disease. We have the history of at least six patients, who have submitted to ovariotomy, and no tumor was found, but the uterus and its appendages were healthy. But gaseous distention of the intestines very often strongly resembles an abdominal tumor. Dr. Buckner, in a note to a manuscript lecture, observes: "I have been consulted recently by a patient in this city, who supposed, and had been told by her attending physicians, that she labored under ovarian disease. She had formerly been pronounced pregnant. The abdomen was very much distended, almost to the size of the full period of gestation; and her gait was that of a pregnant woman. On examination, however, the uterus was found to be healthy, and the abdomen greatly tympanitic. This tympanitis has entirely disappeared, and the patient, under the use of brisk purgatives, is now as small as ever she was."

The resonance on percussion is an infallible diagnostic sign of this disease; but sometimes a peculiarity arises, and must be guarded against; and that is that the ovarian cyst itself may be tympanitic from its adhesion and subsequent communication with the intestines, the air of the latter passing freely into the cavity of the former; but before this takes place, sufficient time is afforded to discover the nature of the original disease.

8. *From the enlargement of the viscera of the abdomen.* The hardened masses frequently found in multilocular cysts, have been mistaken for the diseases and enlargement of various organs of the body, as the liver, spleen, omentum, mesentery, etc. But when the viscera are enlarged, the general health suffers materially; and the enlargement commences from above, passing downward. The irregularities of an ovarian cyst are only to be found some considerable time after the disease commences; while morbid enlargements of the viscera can be detected from the beginning.—*Cincinnati Med. Observer.*

